# The relationships between symptom severity post COVID-19, stress, social support and adaptation in patients with COVID-19 after discharge from the hospital

**DOI:** 10.1371/journal.pone.0327825

**Published:** 2025-11-03

**Authors:** Panyasuda Yeetho, Suchira Chaiviboontham, Bualuang Sumdaengrit

**Affiliations:** 1 Master’s student, Master of Nursing Science Program (Adult and Gerontological Nursing), Ramathibodi School of Nursing, Faculty of Medicine Ramathibodi Hospital, Mahidol University, Bangkok, Thailand; 2 Ramathibodi School of Nursing, Faculty of Medicine Ramathibodi Hospital, Mahidol University, Bangkok, Thailand; PLOS, UNITED KINGDOM OF GREAT BRITAIN AND NORTHERN IRELAND

## Abstract

**Objective:**

This descriptive research aims to investigate the relationship between symptom severity of COVID-19, stress, social support, and adaptation in patients with COVID-19 after discharge from the hospital.

**Background:**

COVID-19 is a respiratory infection with varying symptoms and severity, requiring tailored treatment based on individual needs. The virus spreads via respiratory secretions within 1–2 meters, requiring isolation to prevent transmission. Social distancing, work disruptions, and school closures cause psychological stress, disrupt routines, alter roles, and increase dependence on others. Roy’s adaptation theory was employed as the conceptual framework to determine the relationship between perceived symptoms severity post-COVID-19, stress, social support, and the adaptation of COVID-19 patients after being discharged from the hospital.

**Methods:**

The sample involved 150 patients with COVID-19 after discharged from the university hospital. Five instruments were employed to collect data, including 1) Personal and health information questionnaire, 2) Symptoms and perceived symptoms severity post COVID-19 assessment form, 3) Stress assessment form, 4) Social support assessment form, and 5) Four aspects of adaptation assessment form for COVID-19 patients The data were analyzed using descriptive statistics and Spearman Rank Correlation coefficient.

**Results:**

The results showed that 81.30% of the sample reported post-COVID symptoms. The symptoms severity was perceived at a low level. The most frequently reported symptoms were coughing (72.00%), phlegm (69.30%), and fatigue (60.70%). The study revealed stress at an adaptive level and attained social support at a moderate level. The perception of symptom severity and stress had a statistically significant negative relation with to post-COVID adaptation (r = −.355, p < 0.01; r = −.413, p < 0.01, respectively, while social support had a statistically significant positive relation with post-COVID adaptation (r = .518, p < 0.01).

**Conclusion:**

The results demonstrated a significant relationship between selected factors and patient adaptation. These findings can inform strategies to manage symptoms and stress, while strengthening social support within discharge planning for patients recovering from COVID-19, thereby facilitating their post-discharge adaptation.

## Introduction

Coronavirus disease 2019 (COVID-19) is a respiratory infection caused by the coronavirus SARS-CoV-2, a pathogen that causes symptoms such as fever, cough, and, in severe cases, pneumonia [1,2]. Common symptoms of COVID-19, such as fever, dry cough, and fatigue, can significantly impact patients by causing physical discomfort, weakness, and difficulty performing daily activities. Severe cases may lead to respiratory distress, prolonged recovery, and potential complications, affecting overall health and quality of life [2,3]. COVID-19 presents diverse symptoms across systems, including respiratory (cough, dyspnea), neurological (headache, anosmia, ageusia), gastrointestinal (nausea, diarrhea), musculoskeletal (joint pain, myalgia), and cardiovascular (palpitations, chest pain) manifestations [1,2,4]. Fatigue, hair loss, and mental health issues, such as depression and anxiety, are also found [5]. Symptom variability and severity necessitate individualized treatment approaches to mitigate health and lifestyle impacts [6]. According to spread pattern, Coronavirus spread through respiratory secretions through the air within a range of 1–2 meters, highlighting the need to isolate patients to prevent further transmission of the infection [7–11]. Avoidance of close contact with anyone by stopping working becomes necessary, imposing restrictions on various activities. Consequently, COVID-19 infection impacts physical health, potentially altering one’s lifestyle and mental well-being [12–14].

Disrupting daily routines, keep distancing from family and friends, stop working without compensation, and closing school induced significant mental stress, thereby contributing to psychological disturbances in patients with COVID-19 [15]. Additionally, social isolation impacts the roles and responsibilities of individuals, increasing their dependence on others by limiting their ability to fulfill work, social, and familial obligations. This can lead to a loss of autonomy, reduced self-efficacy, and increased reliance on others for emotional, financial, or practical support. Adhering to social distancing and being unable to fulfill work or societal roles due to infection prevention restrictions can lead to feelings of uncertainty and a diminished self-image [16].

Providing information is a social support that patients require, including accurate guidance for self-care. Receiving accurate and appropriate information to manage their health can assist patients in alleviating anxiety, enhancing their understanding of the disease, and improving their ability to care for themselves, ultimately leading to better recovery outcomes [17]. COVID-19 patients undergo various adaptations in response to their condition. Physiologically, they manage common symptoms such as fever, cough, and sore throat while routinely using ATK tests to monitor their health status. Upon diagnosis, their self-concept shifts as they adopt protective measures, such as wearing masks and maintaining distance, to safeguard others. The role-function changes are evident as many transition from in-person work to remote work through online platforms due to their inability to attend the workplace. Additionally, in the interdependence mode, patients often rely on co-workers or family members to provide essential support, such as delivering food, during their period of self-isolation.

Adaptation arises from an individual’s coping mechanisms when faced with life events or changes. According to Roy’s theory [18], adaptation in patients is influenced by various stimuli. For COVID-19 patients, the infection serves as a focal stimulus, initiating the need for adaptation as the virus spreads and affects the body. The recovery process after infection involves contextual stimuli, such as lingering symptoms, influencing the patient’s adaptation. Residual stimuli, including internal, external, and environmental factors like stress and social support, further impact the patient’s ability to adapt.

In this study, COVID-19 patients faced the need to adapt after infection. Contextual stimuli, such as lingering symptoms post-treatment, influenced their adaptation process. Residual stimuli, including internal factors, external influences, and social support, also played a role in their adaptation. These stimuli triggered various modes of adaptation, including physiological, self-concept, role function, and interdependence modes, helping patients adjust to life after the infection [19]. Sasang [20] study on adaptation in patients with Systemic Lupus Erythematosus (SLE), a chronic condition distinguished by fluctuating symptoms, required patients to employ coping strategies to manage the continual changes in their health status. Common symptoms in SLE patients include edema and obesity, often resulting from renal system abnormalities and medication side effects. The unpredictable onset of the disease can lead to significant stress. Studies on behavioral adjustment programs for SLE patients highlight the importance of providing information on stress management and coping mechanisms to facilitate adaptation during disease relapses [20]. Similarly, following COVID-19 infection, the unpredictable emergence of symptoms may induce stress which require patients to adapt to various stimuli and challenges.

Emerging diseases pose significant challenges, particularly in understanding how humans, systems, and behaviors adapt to these crises. Roy provides a valuable framework for exploring these dynamics, focusing on the interplay of individuals and systems with their environments to maintain health and equilibrium. While Roy emphasizes individuals’ ability to adapt through physiological, self-concept, role, and interdependence modes, there’s limited understanding of how these processes operate during the uncertainty of emerging diseases like COVID-19. Effective disease response relies on adaptability. This study explores how recovered COVID-19 patients adjust to daily life. It examines perceived symptom severity, stress levels, social support, and their impact on adaptation to improve post-discharge care and support societal reintegration.

In this study, data collection targeted variables directly aligned with the objectives: perceived symptom severity post-COVID-19, stress levels, social support, and adaptation. Symptom severity reflects ongoing physical challenges affecting daily life; stress indicates the psychological burden that may hinder adaptation; social support represents external resources aiding coping and reintegration; and adaptation, measured across the four modes of Roy’s model, reflect overall adjustment to post-COVID-19 life. These data are essential for testing the hypothesized relationships and informing post-discharge care strategies.

Research question: Are there any relationships between perceived symptoms severity post-COVID-19, stress, social support, and the adaptation of COVID-19 patients after being discharged from the hospital?

Research objective: To determine the relationship between perceived symptoms severity post-COVID-19, stress, social support, and the adaptation of COVID-19 patients after being discharged from the hospital.

### Research hypothesis

1. Perceived symptom severity post-COVID-19 and stress have a negative relationship with the adaptation of COVID-19 patients after being discharged from the hospital.
2. Social support has a positive relationship with the adaptation of COVID-19 patients after being discharged from the hospital.

## Materials and methods

### Study design and participants

This correlational descriptive study employed purposive sampling to select participants among COVID-19 patients aged 18–59 who received treatment and were discharged from the hospital. Data collection occurred between

July 1, 2022, and November 30, 2022. The sample size of 138 individuals was determined using G*power [20], with a medium effect size (0.3), α = 0.05, and power = 0.95. An additional 10% was included for potential attrition, resulting in a sample group of 150 individuals.

### Setting

During the pandemic, this university hospital implemented specific adaptations to its care areas and processes for COVID-19 patients. The care process included initial assessment at the Acute Respiratory Infection (ARI) clinic for COVID-19 detection, the establishment of cohort wards and intensive care units for COVID-19 patients, and follow-up phone calls on the first day after discharge, as well as on days 7 and 14, to assess the patient’s ability to self-care at home. Additionally, a home isolation guideline was developed for patients. Data collection from treated patients who returned home was conducted through telephone interviews and Google Forms.

### Instruments

1) The Personal and Health Information Questionnaire, developed by the researcher, comprised closed-ended and open-ended questions covering demographics and household details. Meanwhile, the health information includes brief open-ended queries on symptom onset, treatment, discharge dates, weight, height, body mass index, and underlying disease.
2) The Symptoms and Perceived Symptom Severity Post-COVID-19 Assessment Form, developed by the researcher through a literature review, evaluates persistent post-COVID symptoms following treatment and discharge. While most COVID-19 patients experience mild to moderate symptoms and recover without hospitalization [8], this tool assesses 20 symptoms identified in the literature. It also measures the perceived severity of these symptoms, categorized into five levels based on Best’s framework [21], rated on a 5-point Likert scale (0 = No symptoms to 4 = Most severe). The total severity score ranges from 20 to 80 points, with a score of 0 indicating no symptoms, and higher scores reflecting greater severity.
3) The Stress Assessment Form utilized the ST-5 instrument, authorized by the Department of Mental Health, Ministry of Public Health [22,23]. It assesses feelings of worry or anxiety over the past 2–4 weeks through 5 items scored from 0 to 3 (0 = None/almost none to 3 = Always). Total scores range from 0 to 15 and are categorized as follows: 0–4 = minimal to no anxiety, with effective coping abilities; 5–7 = potential stress or discomfort, requiring adjustment time; ≥ 8 = high anxiety levels potentially impacting physical health, necessitating consultation with healthcare professionals.
4) The Social Support Assessment Form: modified from Elderly Social Support Scale [24] with permission, evaluates social support among COVID-19 patients. Aligned with House’s guidelines, it assesses material, informational, behavioral, and emotional/social support [23] across 21 items. Patients rated each item on a 5-point Likert scale, indicating their level of agreement from 5 = strongly agree to 1 = strongly disagree; the score of negative questions was reversed. This tool supports in assessing the diverse levels of social support during patients’ recovery. The Scores range from 21 to 105, with lower scores indicating limited support and higher scores indicating more substantial support.
5) The Four Aspects of Adaptation Assessment Form for COVID-19 patients was adapted with permission from Sasang’s SLE patient adaptation behavior assessment [20]. It employs a Likert Rating scale of 5 levels, with 1 = not at all indicating lacking that ability or feeling entirely, and 5 = most signifying the highest ability or most frequent feeling. The modified version comprises 42 questions across physical (7 questions), cognitive (12 questions), role function (11 questions), and dependence adaptation assessments (12 questions). Total scores range from 42 to 210 points, with higher scores indicating better adaptation ability. The scores of negative questions were reversed.

### Ethical consideration

The ethics was approved by the Human Research Ethics Review Committee (COA.MURA2022/330) and follows the guidelines of the Helsinki Declaration; patients were invited to participate in the study and provided written consent after being informed of the research objectives, benefits, and risks. Participation was voluntary, with the option to withdraw at any time without affecting their treatment. All participant information was kept confidential, and they were encouraged to contact the researchers with any inquiries. The dataset from this study is not publicly available to protect participants’ privacy, in strict compliance with Institutional Review Board (IRB) requirements and data protection regulations. It contains sensitive health and demographic information. In line with IRB guidelines, individual-level data are reported only in aggregate form. Public release of the raw dataset would breach participant consent and institutional confidentiality agreements. De-identified data that pose no privacy risk, along with a data dictionary, may be available upon reasonable request to the corresponding author, subject to review for compliance with ethical approval and data use agreements. This policy ensures transparency while upholding ethical responsibilities, in accordance with international standards for responsible health research data sharing.

### Data analysis

Descriptive statistics [25], including percentage, average, minimum-maximum, frequency, and standard deviation, were used to analyze personal information, symptom severity post-COVID-19, stress, social support, and adaptation of COVID-19 patients. The correlation between post-COVID-19 symptom severity, stress, social support, and adaptation after hospital discharge was also examined. The Kolmogorov-Smirnov test indicated a non-normal distribution for those variables. Consequently, the Spearman rank correlation coefficient was used for the analysis.

## Results

The participants comprised 150 patients with COVID-19, ages 19–59 years old, with a mean of 34.10 (S.D. = 9.99). Most of them were female (68.00%), single (65.30%), and held a bachelor’s degree (52.70%). In terms of monthly income, the majority had sufficient income (74.00%). Most of the samples lived in a house (40.70%) with the family role as a member (73.3%) and a head of the family (26.70%)—the residents’ relations mainly with family members 68.70%, as shown in Table 1.

**Table 1:** Participant Characteristics (N = 150).

Personal information	N (%)
**Gender**	
Female	102 (68.00)
Male	48 (32.00)
**Age**: Min = 19, Max = 59, Mean = 34.10, S.D. = 9.99	
**Marital status**	
Single	98 (65.30)
Married	43 (28.70)
Widowed	4 (2.70)
Divorced/Separated	5 (3.30)
**Educational level**	
Primary education	10 (6.70)
Secondary education	25 (16.70)
Diploma degree	13 (9.40)
Bachelor’s degree	79 (52.70)
Postgraduate degree	22 (14.70)
**Occupation**	
Others (Civil servants, general contractors, company employees)	56 (37.30)
Healthcare provider	45 (30.00)
Merchant	23 (13.30)
Unemployed	12 (8.00)
Teacher	5 (3.30)
Engineer	4 (2.70)
Agriculture/Fishery/Cattleman	2 (1.30)
Architect	2 (1.30)
Accountant	1 (0.70)
**Income/Month (Baht)**	
5,000^–^10,000	24 (16.00)
10,001^–^20,000	45 (30.00)
20,001^–^30,000	41 (27.30)
30,001^–^40,000	20 (13.30)
40,001^–^50,000	8 (5.30)
More than 50,000	12 (8.00)
**Adequacy of income**	
Enough	111 (74.00)
Not enough	39 (26.00)
**Type of Accommodation**	
House	61 (40.70)
Townhome/Commercial building	31 (20.70)
Apartment	25 (16.70)
Condo	19 (12.70)
Others (Not specified)	14 (9.30)
**Role in family**	
Head of the family	40 (26.70)
Family member	110 (73.30)

According to the data collection on all 20 symptoms, it was found that 81.30% of post-COVID-19 infection patients still had symptoms and 18.70% of those who were asymptomatic. There was a low level of perception of the severity of the symptoms post COVID-19. The mean perceived symptoms severity was found to be between 0.13–1.47. Most of the remaining symptoms were coughs 72.00%, phlegm 69.30%, fatigue 60.70%, dyspnea 54.00%, and fever 54.00%. Overall, the perceived severity of symptoms post-COVID-19 is mild. The data are shown in Table 2.

**Table 2:** Descriptive report the perceived symptoms severity post-COVID-19 in the participants. (N = 150).

Symptom	Symptom Frequency	Perceived symptoms severity			Level of severity
**Number (%)**	**Mean**		**S.D.**	**Median**
Cough	108 (72.00)	1.47	1.28	1.00	Mild
Phlegm	104 (69.30)	1.36	1.20	1.00	Mild
Fatigue	91 (60.70)	1.23	1.30	1.00	Mild
Dyspnea	81 (54.00)	1.10	1.27	1.00	Mild
Fever	81 (54.00)	0.97	1.11	1.00	Mild
Muscle pain	74 (49.30)	0.97	1.20	0.00	Mild
Headache	67 (44.70)	0.87	1.14	0.00	Mild
Hair loss	66 (44.00)	0.99	1.36	0.00	Mild
Sleeping problems	62 (41.30)	0.86	1.24	0.00	Mild
Taste changes	61 (40.70)	0.80	1.17	0.00	Mild
Smell changes	59 (39.30)	0.86	1.26	0.00	Mild
Diarrhea	51 (3400)	0.57	0.95	0.00	Mild
Lose weight	50 (33.30)	0.58	0.99	0.00	Mild
Dizziness	48 (32.00)	0.63	1.11	0.00	Mild
Palpitations	36 (24.00)	0.45	0.93	0.00	Mild
Sensitive skin	36 (24.00)	0.49	1.02	0.00	Mild
Chest pain	34 (22.70)	0.43	0.91	0.00	Mild
Discomfort or itchy skin	34 (22.70)	0.47	1.03	0.00	Mild
Rash on the body	27 (18.00)	0.29	0.75	0.00	Mild
Conjunctivitis	24 (16.00)	0.24	0.62	0.00	Mild
Other symptoms	14 (9.30)	0.13	0.50	0.00	Mild
(Specify numb hands, sore throat, and excessive sweating)					

**Overall perceived symptoms severity post COVID-19: Mild; Mean = 15.82,**

**S.D. = 14.92, Median = 11.00.**

**Note: One person has many symptoms.

According to the data on stress characteristics according to the Stress Assessment Form (ST-5), most participants had minimal to no stress, with practical coping abilities, 103 people (68.70%). Thirty (20.00%) people reported potential stress requiring adjustment time, while 17 people, accounting for 11.30%, determined high-stress levels that impacted physical health. The researcher had to assess and refer to the specialist for management. The stress score ranges from 0–15 with a mean of 3.29 (S.D. = 3.54, mode = 0, median = 2)

For social support, the participants had moderate social support with a mean of 72.27(S.D. = 19.92). According to House’s framework [23], the support assessment across four areas showed that facility social support had a high mean score of 18.43 (S.D. = 5.19). Informational social support was also rated highly, with a mean of 20.21 (S.D. = 7.30). Emotional and social support followed closely, averaging 20.05 (S.D. = 4.47). In contrast, appraisal social support was moderate, with a mean score of 13.57 (S.D. = 4.83). These findings are detailed in Table 3.

**Table 3:** Descriptive report of social support (N = 150).

Social support	Possible range	Actual range	Mean	S.D.	Interpret
Facility	5-25	5-25	18.43	5.19	High
Information	6-30	6-30	20.21	7.30	Moderate
Social	6-30	6-30	20.05	4.47	Moderate
Appraisal	4-20	4-20	13.57	4.83	Moderate
**Overall**	**21-105**	**21-105**	**72.26**	**19.92**	**Moderate**

The study of adaptation applied 42 questions, which were divided into four modes according to Roy’s concept of adaptation theory [19]: physiological adaptation, self-concept, role function, and interdependence. The results revealed that the overall adaptation of the participant was at a high level ($\bar{x}$ = 155.45, S.D. = 21.42), and when considering each mode, it was found that the physiological adaptation mode was at a moderate level ($\bar{x}$ = 25.47, S.D. = 4.05). The self-concept mode was high ($\bar{x}$ = 45.09, S.D. = 7.69). The role function mode was high ($\bar{x}$ = 41.35, S.D. = 6.45), and the interdependence mode was moderate ($\bar{x}$ = 43.54, S.D. = 7.12). Details are shown in Table 4.

**Table 4:** Descriptive report of four modes of adaptation (N = 150).

Adaptation mode	Possible range	Actual range	Mean	S.D.	Interpret
Self-concept	12-60	24-60	45.09	7.69	High
Role function	11-55	15-51	41.35	6.45	High
Physiological	7-35	13-35	25.47	4.05	Moderate
Interdependency	12-60	20-57	43.54	7.12	Moderate
**Overall**	**42-210**	**72-203**	**155.45**	**21.42**	**High**

The analysis of correlation data using the Spearman rank correlation coefficient revealed that the perception of symptom severity and stress had a statistically significant negative relation with post-COVID adaptation (r = −.355, p < 0.01; r = −.413, p < 0.01, respectively, while social support had a statistically significant positive relation with post-COVID adaptation (r = .518, p < 0.01), as shown in Table 5.

**Table 5:** The correlation coefficient between the selected factors and the dependent variable.

Variable	Adaptation of COVID-19 patients after discharge from the hospital
Perceived symptoms severity post COVID-19	^–^.355*
Stress	^–^.413*
Social support	.518*

*Correlation is significant at the 0.01 level (2-tailed).

## Discussion

The participants in this study still had post-COVID-19 symptoms (81.30%), with an asymptomatic rate of 18.70%, consistent with the study of the prevalence and nature of chronic symptoms after COVID-19. In western France, there were 311 cases (68.80%) with post-COVID-19 symptoms that remained symptomatic after six weeks of follow-up, and 115 cases recovered (53.7%) [26]. Our study identified 20 symptoms in patients, with cough, phlegm, and fatigue being the most prevalent and severe. Their mean severity scores were 1.47, 1.36, and 1.23 respectively, indicating relatively low levels. These top symptoms were predominantly respiratory, aligning with long COVID-19 patterns post-hospitalization. Furthermore, 54% of participants experienced persistent fever as a long-term symptom, a contrast to previous studies where fever was primarily noted during hospitalization [26,27]. In 2022, post COVID-19 symptoms like fatigue, muscle weakness, chest pain, and respiratory issues were reported [28,29]. These symptoms, though relatively mild, notably affected the respiratory system more than other bodily systems, contrasting with earlier findings.

The differences in symptomatology between the Delta and Omicron variants offer valuable insights into the results of our study, which was conducted during the Omicron variant outbreak. Emerging research has consistently highlighted the unique clinical profiles of these two SARS-CoV-2 variants, each presenting distinct challenges and patterns of disease progression. During the Delta variant surge, individuals often reported prolonged post-COVID-19 symptoms, with data suggesting a significantly longer persistence compared to Omicron. The odds of experiencing lingering symptoms were markedly higher during the Delta period, ranging from 0.24 to 0.50 depending on age and vaccination status [30]. This prolonged recovery contrasted sharply with the Omicron wave, where post-acute sequelae appeared to be shorter-lived.

Symptom prevalence also differed between the variants, as shown in studies conducted in the United Kingdom. For instance, loss of smell—a hallmark symptom in earlier COVID-19 waves—was far less common during the Omicron outbreak, affecting only 16.7% of cases compared to 52.7% during the Delta period. This significant reduction (OR 0.17; 95% CI 0.16–0.19, p < 0.001) reflects Omicron’s departure from some of the more debilitating sensory impacts seen with Delta. Conversely, sore throat, while prominent in both waves, was reported more frequently during the Delta outbreak (70.5%) than Omicron (60.8%), with an odds ratio of 1.55 (95% CI 1.43–1.69, p < 0.001). Hospitalization rates, another critical metric, were lower during the Omicron period. In the Delta wave, hospital admissions reached 2.6%, while Omicron saw a reduction to 1.9% (OR 0.75; 95% CI 0.57–0.98, p = 0.03), underscoring the milder disease course associated with Omicron infections [30,31]. Additionally, research from Mexico highlighted stark contrasts in asymptomatic rates. Omicron infections were asymptomatic in 23.3% of cases—a significant increase compared to Delta (4.4%) and the Wild-Type SARS-CoV-2 (1.9%). Omicron was also characterized by a predominance of milder symptoms, with sore throat and runny nose being the most common, while Delta and earlier variants were associated with more severe presentations [32]. These findings illustrate a clear shift in the clinical profile of COVID-19 with the emergence of Omicron. While the Delta variant posed greater challenges with more severe symptoms and prolonged recovery, Omicron’s milder presentation, coupled with higher asymptomatic rates and reduced hospitalizations, marked a turning point in the pandemic’s trajectory.

Our study evaluated post-COVID-19 symptom severity, categorizing cases into asymptomatic, mild, moderate, or severe based on the World Health Organization’s (WHO) guidelines for assessing the severity of COVID-19 infections [33]. Most participants in our study reported experiencing mild symptoms. This observation aligns with findings from multiple reports, which consistently indicate that over 80% of COVID-19 cases globally present with mild symptoms [34–36]. Such concordance reinforces the understanding that the majority of infections, particularly in certain populations, manifest as less severe clinical presentations.

The statistical analysis revealed a significant negative correlation between post COVID-19 symptoms and patients’ adaptation post-discharge from the hospital.

According to Roy’s adaptation theory, successful adaptation depends on both the stimulus and the individual’s level of adaptation. Symptoms severity after COVID-19 act as contextual stimuli. To maintain balance in life, individuals must adapt their four modes of adaptation. This study revealed high adaptation levels in self-concept and role function modes, while physiological and interdependence modes showed moderate levels of adaptation. This could be explained by the fact that the adaptation pattern may be attributed to the body’s physiological response to infection, wherein symptom severity influences the adaptation process.

In this study, high scores in self-concept mode adaptation indicate that patients perceived the severity of symptoms and adjusted their self-concept by using cognate mechanisms. Thirty-five patients had to stop working and isolate due to COVID-19, impacting their role-function mode. Despite this, a high level of adaptation in role function was observed, likely influenced by the implementation of work-from-home guidelines during the outbreak, which facilitated patients in adjusting their roles and responsibilities.

The study found moderate adaptation levels in the interdependent mode. This suggests that when patients encountered COVID-19 symptoms, they adjusted their self-concept, roles/responsibilities, and interdependence, influenced by factors such as perceived severity, cognitive processes, and situational guidelines during the outbreak. The moderate adaptation in the interdependent mode may be attributed to the fact that 31.30% of participants lived apart from their families during mandated COVID-19 home quarantine, affecting support for housing and food. Illness and fatigue pose challenges to fulfilling roles and maintaining self-confidence. However, rest is essential for recovery, even though it involves isolation and dependence on others for basic needs to regain balance.

The severity of COVID-19 symptoms affects psychological and functional adaptations across self-concept, roles, and interdependence, influenced by factors such as living situation, illness experience, and quarantine guidelines. Lifestyle changes due to the COVID-19 outbreak, such as social distancing and quarantine measures, have had significant impacts. Studies have examined the mental health effects, revealing that 41.4% of 70 COVID-19 survivors experienced mild depression, while 32.9% reported no mental effects after isolation and symptoms [37]. In a recent study in China, 1,210 individuals from 194 cities were surveyed 14 days post-infection during a severe outbreak. The respondents reported a moderate psychological impact, with 53.8% experiencing moderate to severe depression, 16.5% experiencing moderate to severe anxiety, and 28.8% reporting moderate to severe stress levels. This outbreak led to significant lifestyle changes and mental health challenges, including depression, anxiety, and stress, for many COVID-19 patients and survivors [38,39]. Our study findings regarding stress levels among post-hospitalization COVID-19 patients are consistent with previous research. In a sample of 150 cases after hospital discharge, participants demonstrated an adaptive stress level. The analysis revealed a negative correlation between stress and adaptation in post-COVID-19 patients after discharge, indicating that higher stress levels were associated with lower adaptation. This finding aligns with prior research that has shown decreased physical and mental well-being during the pandemic, particularly when adjusting the work from home situations [40].

Even after hospital discharge, COVID-19 infection can induce stress, which can impact physiological adaptation. Achieving physiologic mode adaptation requires balancing between physical and mental states [38]. These findings highlight how stressors from the illness and lifestyle disruptions like isolation/work from home can challenge holistic adaptation and well-being for COVID-19 patients post-hospitalization.

The study revealed a moderate level of overall social support within the sample group. However, scores were notably high for support related to facilities/amenities. This suggests a significant need for such support to assist COVID-19 patients readjust to daily life post-infection. This finding aligns with House’s theory, which includes facilities/amenities as components of social support [24], and Roy’s theory, which emphasizes the importance of care in adapting to the interdependent mode. The findings highlight how tangible resources and services play a crucial role in facilitating patients’ adaptation and transition to interdependence following COVID-19.

The study indicated that social support had a moderate influence on post-COVID-19 adaptation, which aligns with the interdependence mode of Roy’s theory. This suggests that adequate social support plays a crucial role in facilitating adaptation for COVID-19 patients recovering at home. Adaptation is essential for social stability, requiring individuals to find a balanced interdependence – neither dysfunctional, dependent, nor independent. The goal is to appropriately rely on oneself and others within reasonable limits to achieve societal acceptance and live harmoniously. The findings highlight how moderate social support assists this vital interdependent adaptation process for COVID-19 patients transitioning back to community life after infection.

## Conclusion

This study highlights the interplay between the perceived symptoms severity post COVID-19, stress levels, social support, and patient adaptation during the transition back to society after hospital discharge. These findings provide critical insights to guide discharge planning and optimize resource allocation for patients with COVID-19. The observed associations are theoretically supported by Roy’s Adaptation Model, which explains how physiological, psychological, and social stimuli collectively shape patient adjustment. While the study focused on COVID-19, the framework and findings apply to other infectious diseases, such as influenza, pneumonia, and unpredictable emerging diseases, where prolonged symptoms, psychological stress, and reliance on social support impact recovery trajectories. Incorporating these insights into discharge planning could improve continuity of care and community reintegration across diverse patient populations.

These findings provide an evidence-based for enhancing continuity of care beyond hospitalization. The study focused on patients who had been treated for COVID-19 and returned home, suggesting that stress levels in this group may have diminished over time. However, conducting a similar study promptly on stress levels in patients infected with a newly emerging disease could yield different results. This data could then be utilized to enhance planning for appropriate care for such patients during outbreaks. Moreover, a longitudinal study design provides a more comprehensive understanding of changes in symptoms and adaptation among COVID-19 patients post-hospitalization over time. Additionally, promptly studying stress levels in patients with new emerging diseases is recommended, as the findings could inform enhanced care planning tailored to patients’ needs during outbreaks.

### Future research

1. This research is a study of a group of patients infected with COVID-19 who have received treatment and have returned home. Therefore, the effect of anxiety in the patient group may decrease over time. If there is a rapid study of stress among patients infected with emerging diseases, different results may be seen, and information about stress among patients infected with epidemics can be used to plan care more appropriately.
2. The results of this research found that long COVID-19 symptoms, stress, social support, and adaptation of patients infected with COVID-19 when they return home. Therefore, basic information from this study can be used to support the preparation of discharge planning programs for patients infected with other epidemics.
3. Longitudinal research will reveal patterns of symptom change and adjustment more clearly.

## Supporting information

S1 File Instruments. The file contains the instruments used in this study.
(PDF)
