# Supplementary material for: The relationships between symptom severity post COVID-19, stress, social support and adaptation in patients with COVID-19 after discharge from the hospital
Source: PLoS One. 2025 Nov 3;20(11):e0327825. doi: 10.1371/journal.pone.0327825 (PMC12582466; doi:10.1371/journal.pone.0327825)
Supplement: S1 File — The file contains the instruments used in this study. (PDF) [file pone.0327825.s001.pdf]

Supporting Information S1_File. The instruments used in this study.

Instruments

The Personal and Health Information Questionnaire

Part 1 Personal questionnaire

Instructions: Please mark ✓ in the blanks □ or fill in the blanks with the most accurate information.

1. Gender □ Male □ Female
2. Age ………..years
3. Status □ Single □ Married □ Widowed □ Divorced/ Separated
4. Education level

   □ None □ Primary □ Secondary

   □ 2 Years Diploma □ Bachelor's degree

   □ Postgraduate degree Specify … …………………..
5. occupation □ Not working □ Health care □ Merchant

   □ Agriculture/fishery/ livestock

   □ Accounting □ Architect □ Engineer □ Teacher

   □ Other specified........................
6. Income /month □ 5,000 – 10,000 □ 10,001 -20,000 □ 20,001 – 30,000

   □ 30,001-40,000 □ 40,001 – 50,000 □ More than 50,000

   Sufficiency □ enough □ not enough
7. Type of residence: □ detached house □ Townhome / Commercial building

   □ Apartment □ Condo □ Other please specified……………………........
8. Number of people living together…………people
9. Family roles □ Head of family □ Family members
10. Relationships of people living together, □ family □, relatives □ friends

    □ Other, please specified ………………….

Part 2 Health Questionnaire

Instructions: The researcher fills this form based on an interview or health record

1. Date of symptom onset .... Swab date ………….............
2. Date of treatment...
3. Date of discharge from hospital.................................
4. Weight ……… Height ……….. Body Mass Index ....
5. Underlying disease: ☐ None ☐ Yes, please specify the disease ………………………

The Symptoms and Perceived Symptom Severity

Post-COVID-19 Assessment Form

Instruction: Please check ☑ in the box which represents the symptoms of illnesses that existed after discharge within 2 weeks. If choose yes, please assess the severity of the symptom. If no, don't need further assessment.
The scoring criteria are as follows:
A score of 1 means symptoms are mild. A score of 2 means symptoms are moderately severe.

A score of 3 means the symptoms are very severe. A score of 4 means the symptoms are the most severe.

**Symptoms**	**Yes**	**No**	**Symptoms Severity**			
			**1**	**2**	**3**	**4**
1. Fever						
2. Cough						
3. Have phlegm						
4. Difficulty breathing						
5. Headace						
6. Muscle pain						
7. Conjunctivitis						
8. Have a rash on the body						
9. Taste changes						
10. Smell changes						
11. Diarrhea						
12. Uncomfortable itchy skin						
13. Sensitive skin						
14. Hair loss						
15. Tired easily						
16. Have problems sleeping						
17. Weight loss						
18. Chest pain						
19. Palpitations						
20. Dizziness						
21. other						

The Stress Assessment Form (ST - 5)

Instructions: Please put a mark ☑ in the blank ☐ or fill in the blank to match your most feelings by selecting only one answer. There are criteria for selecting answers as follows:

A score of 0 means very little or almost none.

A score of 1 means sometimes, more than once.

A Score 2 means often, almost every day.

A Score 3 means regularly every day.

Number	Symptoms or feelings that have occurred within 2-4 weeks	score			
		0	1	2	3
1	Having sleep problems. Can't sleep or sleep a lot.				
2	Having less concentration.				
3	Irritated, restless, anxious.				
4	Feeling bored and bored.				
5	Don't want to meet people.				
Total score					

Interpretation of results

0 - 4 points means the stress level will not cause problems for oneself. Can still handle the stress that occurs in daily life and adapt to various situations appropriately.

5 - 7 points means may be having stress or a problem causes uncomfortable and has not been resolved, which requires time to adjust or solve the problem. Counseling or advice should be given in terms of relaxing stress by talking or consulting with close people

8 or more means having high levels of stress that may have adverse effects on physical, such as headaches, back pain, insomnia, etc. Consultation from health care provider is required to find the causes of stress and solutions.

The Social Support Assessment Form

Instructions: The following questions describe your experiences over the past 2 weeks. Please checkmark ☑ in the blank about the event or your feelings, which reflect yourself. The answer provides 5 options about receiving support from family, friends, and close people as follows:

Most true means the patient receives the most frequent support.

Very true means the patient receives very much or often support.

Moderately true means the patient receives moderate or occasionally support.

Little true means the patient receives little or infrequent support.

Least true means the patient receives minimal or no support from.

Number	Items	Least true	Little true	Moderately true	Very true	Most true
1	Family members provide help when you have financial problems.					
2	Family members help you with homework, such as cleaning the house, cooking, etc.					
3	Family members take care of you when you need to see a doctor. or travel to various places					
4	Family members arrange accommodation for you as appropriate.					
5	Family members provide clothing and food for you as appropriate.					
6	Family members advise on eating for you.					
7	Family members advise on exercise for you.					

Number	Items	Least true	Little true	Moderately true	Very true	Most true
8	Family members advise on observing various abnormal symptoms.					
9	Family members advise on how to take care of oneself/ how to manage when abnormal symptoms occure.					
10	Family members bring advice from doctors or nurses to you.					
11	When you doubt your health, you can seek advice from family members.					
12	Family members remind you when you become too irritated or stressed.					
13	Family members keep reminding you to see a doctor if something is abnormal with your health.					
14	Family members remind you to go to your doctor's appointments.					
15	Family members warn you when you don't follow the doctor's advice.					
16	You have someone you can trust to consult and talk to you about every problem.					
17	When you are sick or have problems, you usually receive love and care from your family members.					
18	You are confident that you will receive help from those close to you if danger occurs to you .					

Number	Items	Least true	Little true	Moderately true	Very true	Most true
19	You have participated in social activities with friends and relatives.					
20	Family members do not support you to participate in traditional ceremonies or community activities .					
21	Family members encourage you to talk with people who know about health care, such as health care provider.					

The Four Aspects of Adaptation Assessment Form for COVID-19 patients

Adaptation Assessment Form for COVID-19 patients when returning to home

Instructions: By selecting only one answer, please checkmark □ or fill in the blanks that reflect your feelings. The answer provides 5 options as follows:

The Most means you have that feeling the most or regularly.

A lot means you have that feeling a lot or often.

Moderate means you have that feeling moderately or occasionally.

Little means you have that feeling rarely or infrequently.

Not at all means you don't have that feeling at all.

Items		Feeling level				
		the most	a lot	moderate	little	not at all
Physical aspect						
1	You feel easily tired and have difficulty breathing.					
2	You can do various activities normally.					
3	You have a cough until it interferes with normal life					
4	You feel worried when people know you are sick with the COVID–19.					
5	You do not dare to cough or sneeze while there are people around you.					
6	You can return to work normally.					
7	You sleep tightly at night.					
Self-concept aspect						
8	You think your body is perfect and strong.					
9	You are satisfied with your appearance.					
10	You feel discouraged in all aspects of your life.					
11	You feel hesitant and unable to make even the smallest decisions that you once found easy.					
12	You are not sure what will happen to your life.					
13	You still feel proud of yourself even though you are sick / infected with the COVID– 19.					

	Items	Feeling level				
		the most	a lot	moderat e	little	not at all
14	You think that there are others who are more miserable than you.					
15	You are able to accept the illness that has affected you.					
16	You feel that you are still valuable and useful to your family.					
17	You feel guilty about being a burden to others to take care of.					
18	You think that if you had sought treatment sooner, the symptoms wouldn't be as severe.					
19	You feel that your sexual desire has decreased.					
Role function aspect						
20	You are interested in the stories and experiences of your family members.					
21	You can perform your role as a father, mother, child or wife as usual.					
22	You feel that family members do not ask for advice from you like they did before your illness					
23	You still participate in decision-making in the family.					
24	You can play your role as before your illness.					
25	Currently, you have participated in various activities in society as usual					
26	You still work hard as before thus making the disease more severe.					
27	You follow the advice of doctors and nurses regularly.					
28	You take your medicine and come for regular checkups.					
29	When you notice any abnormal symptoms, you promptly see a doctor or report them to the doctor who comes for regular check-ups.					
30	You explored alternative treatment methods, such as decoctions and potions from local doctors.					
Interdependence Aspect						
31	You ask the doctor or nurse about your disease, test results, and treatment you received.					

Items		Feeling level				
		the most	a lot	moderat e	little	not at all
32	When the opportunity arises, you discuss and share experiences about illness with others who have been infected with COVID-19.					
3 3	You received good help from family members.					
34	You are satisfied with the relationships in the family.					
35	You feel warm when you are with your family.					
36	You prefer to have others assist you with everything.					
37	You felt that your life was definitely dependent on others.					
38	You will undertake various activities on your own, even if some tasks are beyond your ability.					
39	You can provide assistance to others as requested.					
40	You know the various resources available to you for help.					
41	You seek help by talking or venting your feelings to relatives, nurses, or patients.					
42	Your family members are knowledgeable about COVID-19 and are promptly to help you.					
